# Supplementary material for: AAV8-based gene replacement therapy for hereditary spastic paraplegia type 5
Source: Mol Ther Methods Clin Dev. 2025 Jul 15;33(3):101531. doi: 10.1016/j.omtm.2025.101531 (PMC12309954; doi:10.1016/j.omtm.2025.101531)
Supplement: Document S1. Figure S1 [file mmc1.pdf]

OMTM, Volume *33*

Supplemental information

AAV8-based gene replacement therapy for hereditary spastic paraplegia type 5

Linus Wiora, Qinggong Yuan, Sebastian Hook, Melanie Kraft, Ingemar Björkhem, Michael Ott, Stefan Hauser, and Ludger Schöls

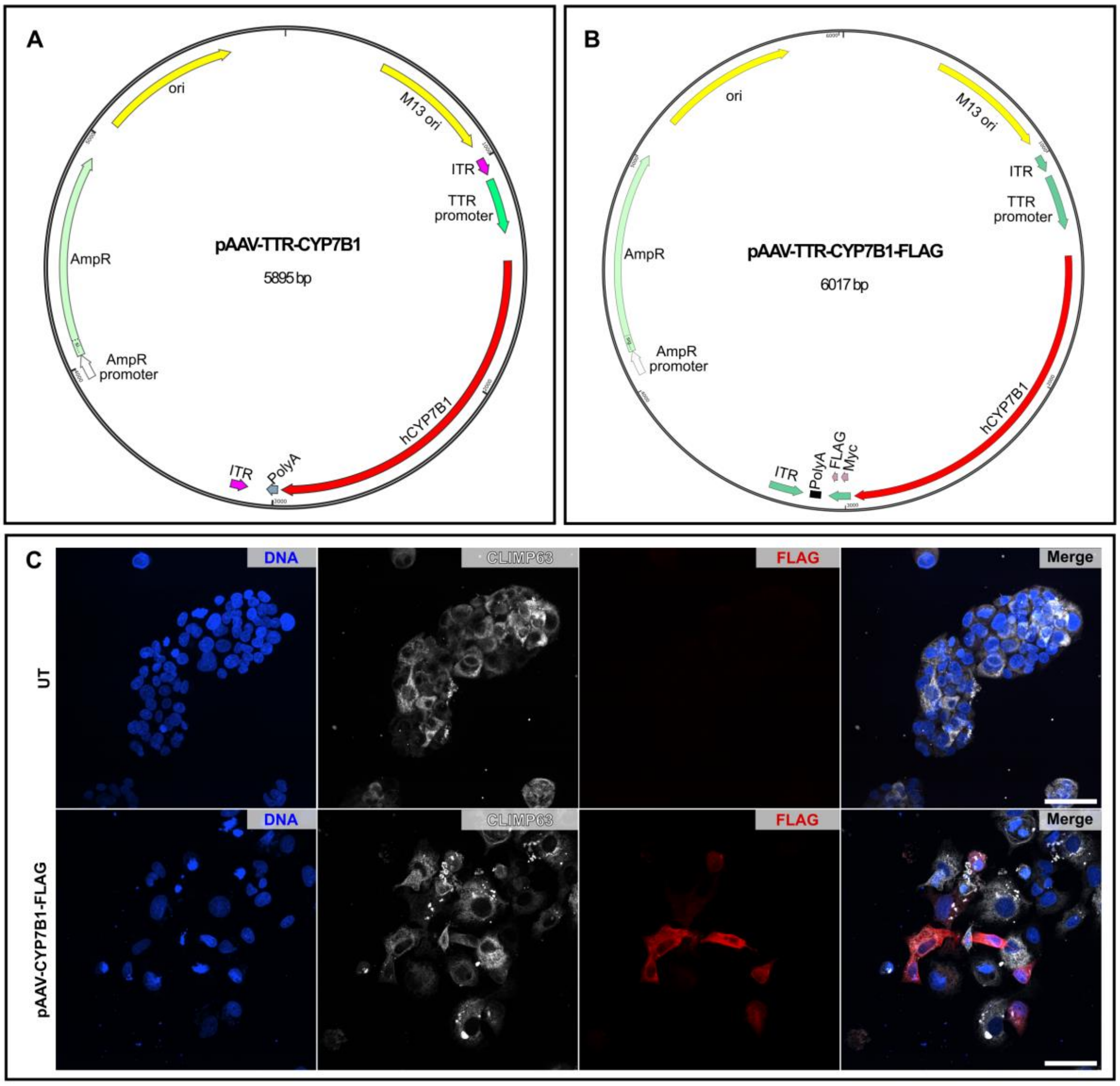

Figure S1: Plasmids and specificity test for FLAG antibody. Plasmid maps of (A) pAAV-TTR-hCYP7B1 and (B) pAAV-TTR-CYP7B1-FLAG construct used for expression test and virus production. (C) HepG2 cells immunostained for the FLAG-tag. Untreated control (UT) shows only signal for the endogenous ER marker CLIMP63, while cells transduced with pAAV-TTR-CYP7B1-FLAG show clear signal localizing in the ER with CLIMP63, demonstrating specificity of the antibody. Scale bar= 50 µm.
