# Supplementary material for: AAV8-based gene replacement therapy for hereditary spastic paraplegia type 5
Source: Mol Ther Methods Clin Dev. 2025 Jul 15;33(3):101531. doi: 10.1016/j.omtm.2025.101531 (PMC12309954; doi:10.1016/j.omtm.2025.101531)
Supplement: Document S2. Article plus supplemental information [file mmc2.pdf]

Molecular Therapy
Methods & Clinical Development
Original Article

AAV8-based gene replacement therapy for hereditary spastic paraplegia type 5

Linus Wiora,[1,2,3,4] Qinggong Yuan,[5] Sebastian Hook,[5] Melanie Kraft,[2] Ingemar Björkhem,[6] Michael Ott,[5] Stefan Hauser,[1,2,4,7] and Ludger Schöls[1,2,4,7]

[1]German Center for Neurodegenerative Diseases (DZNE), 72076 Tübingen, Germany; [2]Department of Neurology and Hertie Institute for Clinical Brain Research, University of Tübingen, 72076 Tübingen, Germany; [3]Graduate School of Cellular and Molecular Neuroscience, University of Tübingen, 72076 Tübingen, Germany; [4]Gene and RNA Therapy Center (GRTC), Faculty of Medicine University Tübingen, 72076 Tübingen, Germany; [5]Department of Gastroenterology, Hepatology, Infectious Diseases and Endocrinology, Hannover Medical School, 30625 Hannover, Germany; [6]Department of Laboratory Medicine, Karolinska Institutet, 171 77 Stockholm, Sweden

Hereditary spastic paraplegia type 5 (SPG5) is an autosomal recessive neurological disorder caused by mutations in the CYP7B1 gene, which encodes cholesterol 7α-hydroxylase, an essential enzyme in cholesterol metabolism. These mutations lead to elevated levels of 25- and 27-hydroxycholesterol, oxysterols known to be neurotoxic and blood-brain-barrier permeable. Their accumulation contributes significantly to SPG5 pathogenesis, resulting in spastic gait disturbance and severely impaired quality of life. Using a Cyp7b1$^{-/-}$ mouse model that mirrors the metabolic phenotype of SPG5, we developed a gene therapy approach to correct oxysterol imbalance. We designed an AAV8-TTR-hCYP7B1 vector to deliver the CYP7B1 gene specifically to the liver. Following intravenous administration, oxysterol levels in blood and liver were rapidly normalized, even at low doses (1E10), with no observed toxicity at the highest tested dose (1E11). Despite these promising peripheral results, oxysterol levels in the brain, particularly 27-hydroxycholesterol, remained only partially corrected six weeks post-treatment. Our findings suggest that while liver-targeted gene therapy is effective at restoring peripheral cholesterol metabolism, a successful therapeutic strategy for SPG5 must also address central nervous system involvement. We conclude that successful treatment of SPG5 would require a novel gene therapeutic approach that also targets the CNS.

INTRODUCTION

The term hereditary spastic paraplegia (HSP) refers to a large group of inherited motor neuron disorders sharing the clinical hallmark of progressive spastic gait disturbance leading to increasing immobility and disability. HSP is primarily characterized by degeneration of the corticospinal tracts in the spinal cord and affects axons of upper motor neurons in a length-dependent manner.[1] Genetically, HSP is highly diverse with so far more than 80 distinct genetic loci mapped to the disease and, more than 50 genes identified spanning all modes of inheritance.[1,2] Affected genes are involved in a wide range of cellular functions including microtubule dynamics (as in spastic paraplegia type 4 [SPG4]), cellular trafficking (e.g., in SPG10, SPG30, and SPG58), and endoplasmic reticulum (ER) membrane shaping (e.g., in SPG3 and SPG31).[3–5]

SPG5 is an autosomal recessive form of HSP with onset in most cases during adolescence (median age of onset: 13 years, range 1–63).[6] Patients suffer from a progressive spastic gait disorder accompanied by afferent ataxia due to sensory deficits prominent in the legs reflecting a predominant involvement of the corticospinal tract and dorsal columns to/from the lower limbs.[7] SPG5 is caused by bi-allelic loss-of-function mutations in *CYP7B1*, which encodes the monooxygenase cholesterol 7α-hydroxylase.[8] This cytochrome P450 enzyme is involved in cholesterol metabolism and essential for the so-called alternative bile acid synthesis pathway.[9] While bile acids can still be synthesized from cholesterol via the so-called primary pathway in the SPG5 situation, the lack of CYP7B1 enzyme activity leads to an accumulation of its substrates 25-hydroxycholesterol (25-HC) and 27-hydroxycholesterol (27-HC), which are formed in the alternative pathway. Accordingly, 25-HC is increased up to 100-fold and 27-HC up to 6-fold in the blood of SPG5 patients.[6,10,11] As 25-HC and 27-HC are side-chain oxidized metabolites of cholesterol that can cross the blood-brain barrier,[12–14] the enhanced levels in serum are regarded the cause of elevated concentrations in the cerebrospinal fluid (CSF) of SPG5 patients.[11] This is of particular importance as hydroxylated cholesterol metabolites have been shown to be neurotoxic and to promote neurodegeneration at concentrations that are close to those found in SPG5 patients.[6,15,16]

The mechanism by which elevated oxysterol levels rather selectively affect the corticospinal tract and posterior column in SPG5 is not fully elucidated. High levels of oxysterols in the brain may lead to increased incorporation of these abnormal cholesterol metabolites

Received 16 May 2025; accepted 9 July 2025;
https://doi.org/10.1016/j.omtm.2025.101531.

[7]These authors contributed equally

Correspondence: Stefan Hauser, German Center for Neurodegenerative Diseases (DZNE), 72076 Tübingen, Germany.
E-mail: stefan.hauser@dzne.de

© 2025 The Author(s). Published by Elsevier Inc. on behalf of The American Society of Gene and Cell Therapy.
This is an open access article under the CC BY license (http://creativecommons.org/licenses/by/4.0/).

into lipid membranes. Since the axons of the corticospinal tract to the legs and the dorsal column fibers from the leg are the longest axons in the human body, the effect of an altered lipid composition of membranes may accumulate in these tracts and may explain their predominant affection.

This led to the hypothesis that lowering 25-HC and 27-HC in the CNS of SPG5 patients may help to slow down or even halt disease progression in SPG5. In a pilot study, we aimed to reduce the levels of 25-HC and 27-HC in SPG5 patients by lowering cholesterol using statins. In a randomized controlled trial, atorvastatin (40 mg/day) reduced blood 25-HC levels by about 20% and 27-HC levels by about 30% after 9 weeks. However, the translation of this treatment effect into the CSF was limited.[6] Even after more intensive cholesterol-lowering treatment with a combination of statins and ezetimibe, oxysterol levels remained much higher than in controls or asymptomatic heterozygous *CYP7B1* mutation carriers.[6,17,18] Given the large gradient between blood and CSF levels of 25-HC and 27-HC in SPG5 patients, a much more efficient reduction of oxysterols is likely required to prevent significant diffusion of 25-HC and 27-HC into the brain.

To this end, we established an mRNA-based enzyme replacement therapy. In a $Cyp7b1^{-/-}$ mouse model[19] with metabolic alterations similar to those of SPG5 patients. Intravenous administration of human *CYP7B1* mRNA-LNP complexes restored liver enzyme activity and strongly reduced blood oxysterol levels. This also helped to partially reduce 25-HC levels in the brain, but cerebral levels of 27-HC remained unchanged.[20] A major drawback of this approach is the limited duration of the therapeutic effect due to the short half-life of *CYP7B1* mRNA and protein. To overcome the limitation of weekly injections and to ensure a long-lasting expression of *CYP7B1*, the present study used a viral vector-mediated gene delivery approach.

Gene therapy using adeno-associated viral (AAV) vectors has made breakthroughs in the last decade, successfully targeting devastating diseases such as spinal muscular atrophy[21] or hemophilia B.[22] Several clinical trials have proven AAV vectors to be an efficient and, most importantly, safe tool to deliver a functional copy of a defective gene to different organs, including the liver (reviewed in a study by Junge et al., 2015).[23] AAV serotype 8 (AAV8) has been successfully used for liver-directed gene transfer and has shown efficacy in clinical trials for metabolic diseases (NCT03517085 and NCT02991144).[24]

Although CYP7B1 is expressed in many tissues, with the highest levels in the brain, we focused on a treatment specific for the liver, because it is the primary source of neurotoxic oxysterols in SPG5.[12–14] Liver-directed gene therapy has already been applied in humans and demonstrated to be both safe and effective, whereas brain-targeted gene therapies are still mostly in early stages of development. Consequently, a liver-directed strategy is proposed as a more efficient and pragmatic approach for the treatment of patients with SPG5. In this study, we evaluated the potential of liver-directed gene therapy for SPG5 using a preclinical mouse model. The human CYP7B1 gene was delivered under the control of the liver-specific transthyretin minimal promoter (TTRmin) via AAV8. Treatment resulted in a rapid and highly significant reduction of oxysterol biomarkers in liver and serum. The effect was sustained until the end of the study, without decay, without side effects or apparent toxicity, and without the need for repeated injections, but was only partially transmitted to the brain.

RESULTS

In vitro validation of h*CYP7B1* construct for AAV vector delivery

In order to test the expression of the generated constructs, the plasmids containing either untagged *hCYP7B1* or *hCYP7B1*-Myc-FLAG under the control of the TTR_{min} promoter were transfected to HepG2 hepatoma cells. GFP-transfected cells served as controls. At 24 h post-transfection, cells appeared healthy and were either harvested for RNA/protein isolation or fixed for immunostaining (Figure 1A). Quantitative PCR showed a 10,000-fold upregulation of *CYP7B1* expression compared to the GFP control, with no obvious difference between the unmodified and the FLAG-tagged construct (Figure 1B). This was confirmed on protein level by western blot, which showed a distinct band at approximately 56 kDa in all samples. While the signal in the TTR-hCYP7B1 and TTR-hCYP7B1-FLAG condition exceeded that of the GFP control (Figure 1C), this was not reflected in the very strong effect observed in qPCR. This can be attributed to a relatively short time (24 h) following transfection during which RNA is translated to protein. Immunostaining of fixed cells against the FLAG tag further confirmed the expression of hCYP7B1 and also the correct subcellular localization in the ER with a strong signal around the nucleus co-localizing with the ER marker CLIMP63 (Figure 1D). The specificity of the anti-FLAG staining was confirmed using a non-transfected control (Figure S1C). For *in vivo* experiments, only the untagged hCYP7B1 construct was used.

Administration of AAV8-TTR-hCYP7B1 significantly reduces oxysterols in serum of $CYP7B1^{-/-}$ mice

To prove the functionality of the generated constructs, $Cyp7b1^{-/-}$mice received intravenous administration of different vector-formulations and dosages. Each experimental group consisted of three male and three female animals to account for sex differences in sterol metabolism. Mice received either one of three doses of the AAV8-TTR-*hCYP7B1* formulation (1E10, 5E10, and 1E11 vg/animal) or the AAV8-TTR-GFP control vector (5E10 vg/animal). In addition, two untreated control groups ($Cyp7b1^{-/-}$ and wild-type $Cyp7b1^{+/+}$ [WT]) were included in the study as reference for oxysterol levels and potential toxicity. Blood samples were collected from all animals seven days prior to viral injection and pooled by group and sex for baseline assessment. An overview of the timeline is shown in (Figure 2A). At baseline, levels of 25- and 27-HC were significantly elevated, whereas 24-HC levels were unchanged in $Cyp7b1^{-/-}$ mice compared to WT controls (Figure 2B).

Overall, the injection was well tolerated. No acute toxicity was observed after injection in any of the experimental groups. The

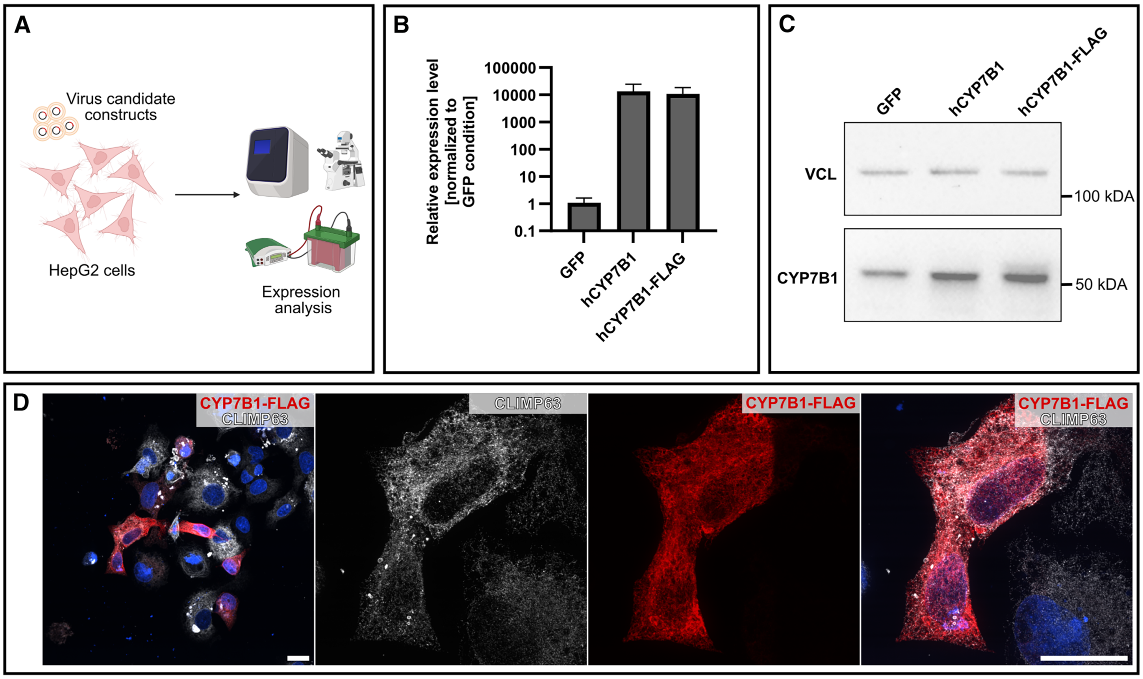

Figure 1. *In vitro* validation of generated constructs

(A) Overview of *in vitro* experiments. (B) Gene expression of CYP7B1 in HepG2 cells after transfection with plasmids for GFP (control), hCYP7B1, and CYP7B1-FLAG assessed by qPCR. CYP7B1 expression after transfection with the respective plasmids is upregulated 10,000-fold compared to untreated and GFP controls. (C) Western blot analysis confirms strong expression of CYP7B1 (VCL, vinculin, loading control). (D) Immunofluorescence of HepG2 cells fixed 24 h after transfection with the CYP7B1-FLAG construct, stained against the FLAG tag, the ER marker CLIMP63, and DNA (scale bars, 20 μm).

animals behaved normally and the injection sites showed no signs of infection or immune response.

Additionally, aminotransferase (aspartate transaminase [AST]/alanine transaminase [ALT]) serum levels were not elevated with any virus dose at the study endpoint. Mean AST values for WT, knockout (KO), and vehicle-treated groups were 62.2 ± 42.4, 87.5 ± 34.9, and 70.8 ± 30.0 U/L, respectively, while in the three treated groups it was 79.3 ± 17.0, 81.8 ± 17.9, and 73.0 ± 23.9 U/L for 1E10, 5E10, and 1E11 vg/animal. For ALT mean control levels were 26.4 ± 3.4, 33.7 ± 15.7, and 27.3 ± 4.9 U/L (WT, KO, and vehicle) and in treated groups 37.0 ± 12.6, 33.2 ± 5.9, and 31 ± 6.2 U/L (1E10, 5E10, and 1E11 vg/animal) (Figure 2C). These values indicate a good safety profile of the designed vector and no apparent hepatotoxicity.

Mice were sacrificed for further analyses of potential toxic and therapeutic effects six weeks after vector application. Blood and organs were collected from each animal for further analyses.

Efficient liver expression of CYP7B1 in Cyp7b1$^{-/-}$ mice after intravenous administration of AAV8-TTR-hCYP7B1

The transduction efficiency of AAV-TTR-hCYP7B1 in the liver was assessed by immunohistochemistry (IHC), qPCR, and western blot. IHC showed widespread expression of CYP7B1 exceeding the WT levels already in the lowest vector dose with increasing expression in the intermediate and high doses. These results were confirmed by qPCR where the human CYP7B1 mRNA could not be detected in control groups, but were expressed similar to the housekeeping gene Actb in the lowest dosage (ΔCt 0.10 ± 0.97). In the highest dose group of AAV-TTR-hCYP7B1 administered animals, the human CYP7B1 expression exceeded Actb by almost 3.5-fold (ΔCt 3.45 ± 0.29). On protein level, the endogenous expression of murine Cyp7b1 could be detected in WT but not in PBS or vehicle injected *Cyp7b1*$^{-/-}$ animals. Even with the lowest vector dose, human CYP7B1 expression exceeded WT expression levels in the liver, whereas the higher dosages led to oversaturation of the membrane with CYP7B1 protein. (Figures 3A–3C). Taken together, a highly effective and dose-dependent expression of the target transgene in the liver of AAV8-TTR-hCYP7B1 injected animals could be validated on transcript and protein level. As a metabolic effect, 25-HC and 27-HC levels in the liver were significantly reduced after treatment. Levels of 25-HC in liver homogenate were no longer different between WT controls and KO animals after treatment in all three dose groups (Figure 3D). Similarly, 27-HC levels were close to WT levels after treatment, especially in the medium- and high-dose group (Figure 3D). In liver tissue, a slight but significant reduction of the cerebral metabolite 24-HC was observed in the two higher dose groups of AAV8-TTR-hCYP7B1 compared

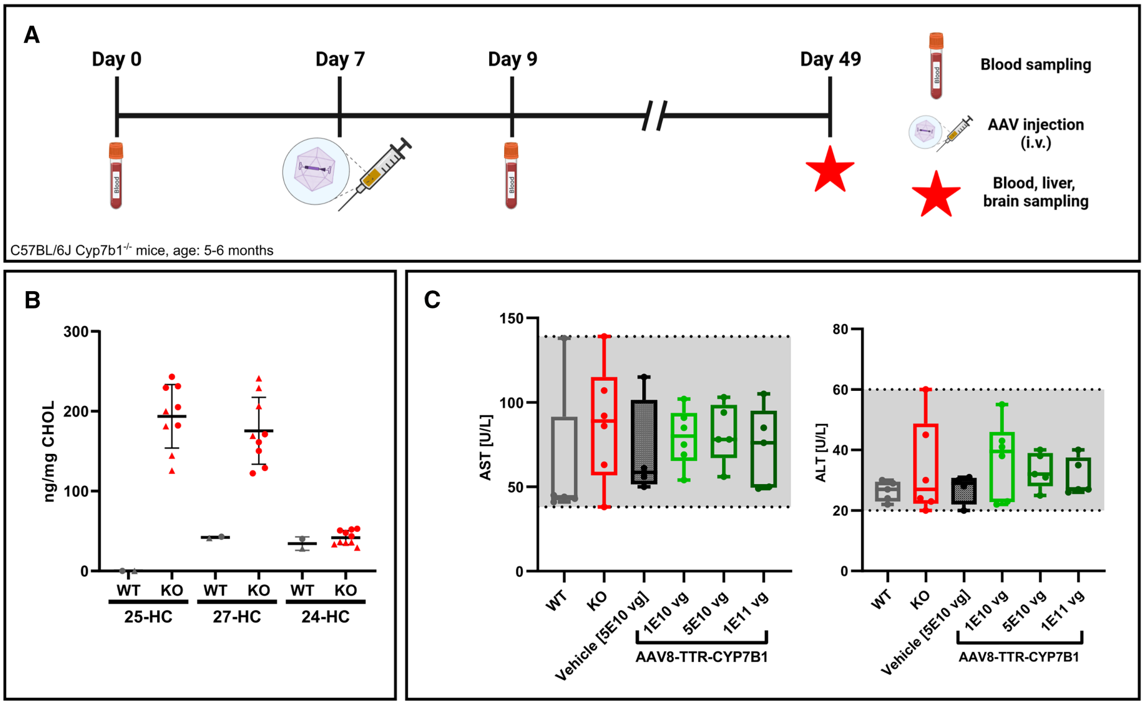

Figure 2. Study design, baseline serum oxysterol levels, and liver toxicity marker

(A) Timeline of animal experiments. Serum was sampled for baseline oxysterol profiling (d0, age between 5 and 6 months) one week before vector administration (day 7) and subsequent blood sampling shortly after treatment (d9) and at study endpoint (d49). (B) Baseline serum levels of oxysterols in WT and KO animals before the study. The biomarkers 25- and 27 HC are significantly elevated in KO animals recapitulating the metabolic phenotype of SPG5 whereas the cerebral metabolite 24-HC remains unchanged (C) Serum AST/ALT levels of animals at the endpoint (D49). No elevation of liver aminotransferases above untreated conditions was observed. Gray background represents range of PBS-treated KO animals.

to the vehicle-treated group (27.7 ± 1.7 ng/mg CHOL in the highest dosage compared to 42.9 ± 5.3 ng/mg CHOL in the vehicle group).

CYP7B1 expression leads to normalization of serum oxysterol levels

Treatment effects in the liver transferred well into blood. After viral injection, oxysterol levels in blood declined rapidly in groups receiving the AAV8-TTR-*hsCYP7B1* construct, but not in the GFP vehicle or PBS groups. Two days after virus administration, the mean serum concentration of 25-HC was reduced close to WT levels with all three vector concentrations (12.3 ± 8.2 ng/mg CHOL in the highest dose group compared to 179.4 ± 40.8 ng/mg CHOL in the vehicle group and undetectable levels in WT animals). The mean serum concentration of 27-HC was also massively reduced and reached levels close to WT animals after treatment with the middle and the high vector concentration (83.8 ± 18.9 ng/mg CHOL in the highest dose compared to 158.3 ± 44.1 ng/mg CHOL in the vehicle group and 42.1 ± 6.2 ng/mg CHOL in WT animals). No relevant change has been observed in serum levels of 24-HC upon treatment (Figure 4A).

The marked reduction in serum 25-HC and 27-HC observed two days post-injection persisted until the endpoint of this study six weeks after injection. 25-HC was substantially reduced to WT levels in the medium and high dose group (1.8 ± 4.5 ng/mg CHOL in the highest dose group compared to 213.2 ± 78.5 ng/mg CHOL in the vehicle group and 0.6 ± 1.5 ng/mg CHOL in the WT controls). Similarly, 27-HC was reduced to near normal levels in the medium and high dose groups (52.7 ± 13.1 ng/mg CHOL in highest dose group compared to 186.0 ± 13.1 ng/mg CHOL in the vehicle group and 50.0 ± 10.1 ng/mg CHOL in WT controls). Again, 24-HC remained unchanged (Figure 4B). No sex-specific differences in oxysterol reduction were observed.

In summary, the differences between vehicle and treatment groups were highly significant at all virus doses for both oxysterol biomarkers (25-HC and 27-HC) with no effect on the cerebral metabolite 24-HC. Compared to WT levels, only the lowest dose of AAV8-TTR-hCYP7B1 did not normalize the serum levels entirely, indicating the lower limit of the therapeutic dose for an effective treatment (Figure 4B).

Systemic reduction of oxysterols in liver and serum only partially translated to the brain

In the brain, the effects of AAV8-TTR-hCYP7B1 treatment on oxysterol levels were clearly different from those in the blood and liver.

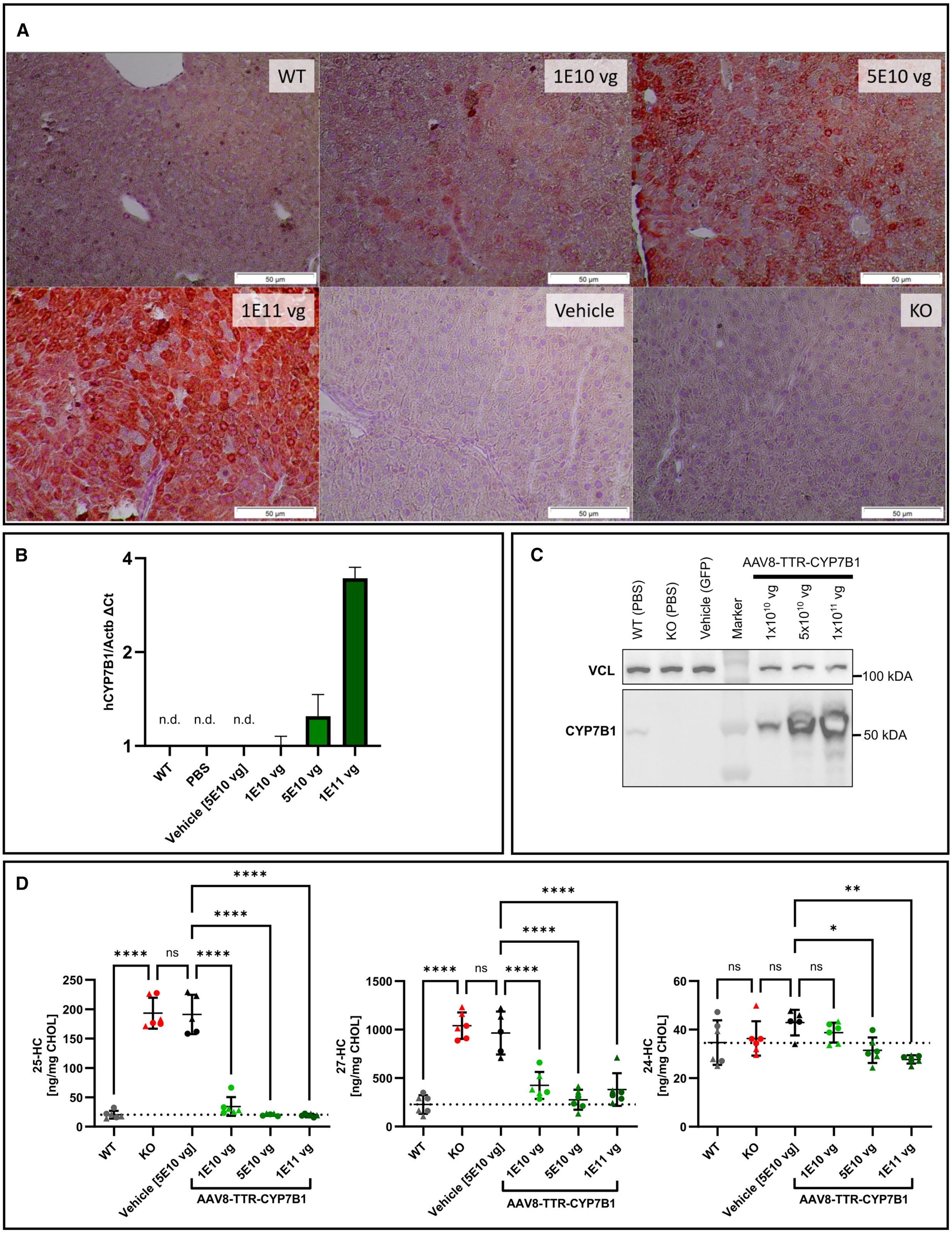

(legend on next page)

The rapid and pronounced decrease of oxysterols in liver and blood only partially translated to the brain (Figure 5). Brain levels of 25-HC declined by 25% but did not reach WT levels (123.3 ± 8.2 ng/mg CHOL in the highest vector dose compared to 157.3 ± 11.9 ng/mg CHOL in the vehicle group and 0.1 ± 0.4 ng/mg CHOL in WT group). No significant changes were observed for 27-HC at any of the vector doses. 24-HC levels remained unchanged except for the highest vector dose, where a slight but significant increase was observed (2665.0 ± 273.9 ng/mg CHOL in the highest vector dose compared to 2258.0 ± 104.3 ng/mg CHOL in the vehicle group and 2471.0 ± 163.2 ng/mL CHOL in the WT group) (Figure 5A).

DISCUSSION

This study demonstrates an efficient, long-lasting correction of key metabolic abnormalities in the liver and blood of Cyp7b1$^{-/-}$ mice by a single intravenous delivery of a human *CYP7B1* gene copy via an AAV8 vector with no evidence of relevant hepatotoxicity or other side effects. The limited reduction of oxysterols in the brain is somewhat surprising and requires further investigation.

Under physiological conditions, it has been shown that the majority of 25-HC and 27-HC enters the brain from the periphery.[13] With elevated serum levels of oxysterols in SPG5, their diffusion or uptake across the blood-brain barrier is likely to be further enhanced. This opens a window for a peripheral/hepatic treatment to modify key metabolic abnormalities in the brain. Since gene replacement in the liver is well established and much easier than in the brain, we aimed for a primary hepatic treatment using an AAV8 vector, which is known to efficiently transduce the liver in both mice and humans. AAV8 has been successfully used in gene therapy trials for metabolic diseases (NCT03517085 and NCT02991144) and bleeding disorders in humans.[22,24] The use of this approved AAV serotype promised to facilitate overcoming the regulatory hurdles of translation and establish a first curative therapy for HSP type 5.

Liver transduction with an AAV8-TTR-hCYP7B1 vector resulted in the expression of functional *CYP7B1* protein exceeding the WT levels even at the lowest dose. Despite the high expression, no acute adverse events were observed and aminotransferases as indicators of liver damage remained within the range of untreated KO animals, suggesting a good safety profile of the treatment. After administration, the newly expressed *hCYP7B1* protein rapidly metabolized the accumulated oxysterols in blood and liver and largely normalized serum concentrations of 25- and 27-HC within two days of vector administration. This indicates rapid expression and high activity of the enzyme soon after injection. The slightly less pronounced effect on 27-HC concentrations compared to 25-HC may be explained by the substrate preference of *CYP7B1* for 25- over 27-HC[25] or a longer half-life of 27-HC. The effect in serum persisted to the endpoint of the study and concentrations of 25- and 27-HC were also normalized in liver tissue, the main source of these oxysterols.[13]

Although the AAV8-TTR-hCYP7B1 vector reduced blood 25-HC and 27-HC levels to near WT levels, somewhat unexpected, this normalization in the periphery had only a limited effect on brain oxysterol levels, despite previous findings that most of 25-HC and 27-HC in the CNS originate from the periphery.[12–14] We observed a similar result in our previous mRNA study that aimed for an mRNA-based enzyme replacement in the liver.[20] In this approach, we attributed the lack of effect in the CNS to the short treatment effect due to the short half-life of the mRNA and CYP7B1 protein, which required frequent re-administration of mRNA. While treatment in the previous study was limited to 17 days, our AAV8-mediated enzyme replacement study lasted six weeks. However, even this treatment period may have been too short. The half-life of oxysterols in the CNS is unknown, but may well be significantly prolonged by their incorporation into membranes. In the case of SPG5, oxysterols may alter the architecture and stability of membranes due to their polar side chain.[26] This may lead to disruption and impairment especially of the longest axonal structures such as the corticospinal tracts to the legs.

An alternative explanation for the limited translation of the peripheral normalization of oxysterol levels to the brain could be an autochthonous synthesis of oxysterols in the CNS. Indeed, sterol 27-hydroxylase, the major enzyme that catalyzes the hydroxylation of cholesterol at position C27, is substantially expressed in the brain where it is involved in the breakdown of the cerebral oxysterol 24-HC.[27] Although the majority of 27-HC in the brain originates from the periphery,[12–14] this limited autochthonous synthesis may be sufficient to maintain elevated levels in the case of defective degradation, as in SPG5 patients and Cyp7b1$^{-/-}$ mice, which lack the key enzyme for its metabolism. Alternatively, the limited amounts of 25-HC and 27-HC that enter the brain under physiological conditions despite low levels in blood may accumulate in the brain in SPG5 as no degradation can take place without CYP7B1 in the CNS. In this case, even permanent normalization of oxysterols in the periphery would not help to normalize CNS levels, but would require restoration of CYP7B1 activity in the brain.

Figure 3. Liver transduction efficacy and liver oxysterol levels

(A) Immunohistochemistry of liver sections against CYP7B1 from wild-type (WT) animals, Cyp7B1$^{-/-}$ animals injected with different doses of AAV8-TTR-hCYP7B1, AAV8-TTR-eGFP (vehicle), and untreated KO animals (KO). (B) mRNA levels of human CYP7B1 and endogenous murine Cyp7b1 for the six experimental groups normalized to Eif2α and Actβ. Strong expression of human CYP7B1 is observed with all doses of AAV-TTR-hCYP7B1. (C) Western blot of liver homogenates (VCL, vinculin, loading control). CYP7B1 expression in all groups receiving AAV-TTR-CYP7B1 injections exceeded WT expression and highest doses led to oversaturation of the membrane, while untreated and vehicle-injected KO animals show no signal for CYP7B1. (D) Liver oxysterol levels at the endpoint of the study (D49). Both 25-HC and 27-HC were significantly reduced compared to the GFP vehicle-treated group. Levels of the cerebral metabolite 24-HC decreased slightly but remained within the range of WT animals. *$p < 0.05$; **$p < 0.01$; ***$p < 0.001$; and ****$p < 0.0001$.

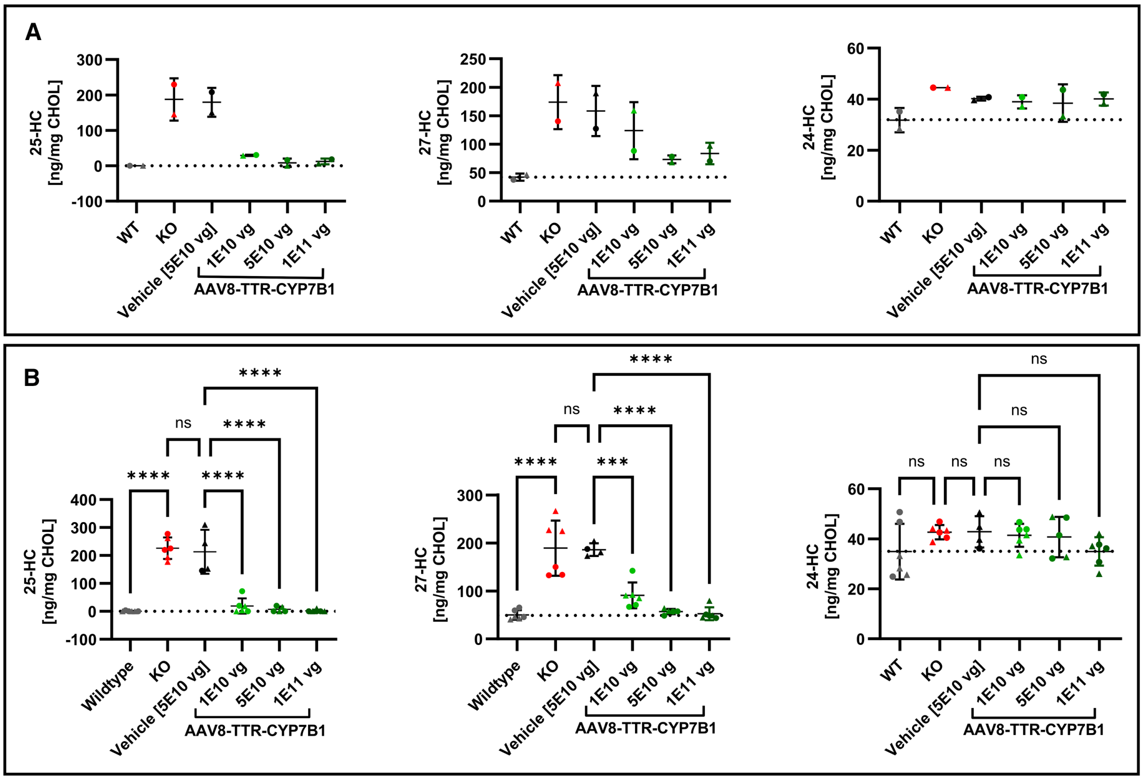

Figure 4. Normalization of blood oxysterols

(A) Serum oxysterol levels of pooled blood samples (sex matched) two days after AAV injection (d9). 25-HC concentrations reached WT level (25-HC) whereas 27-HC levels showed a dose dependent reduction of approximately 20%–50% relative to vehicle group. 24-HC remained unchanged. (B) Serum oxysterol levels of each animal at the endpoint (D49) showed normalization to WT levels for both oxysterols in all vector dosages and except the lowest dose where 27-HC was only reduced by approximately 50% relative to vehicle. Pairwise comparison to AAV8-TTR-GFP vehicle group. ns, not significant; *$p < 0.05$; **$p < 0.01$; ***$p < 0.001$; and ****$p < 0.0001$.

This finding is particularly important when considering liver transplantation as a potential treatment option for CYP7B1 deficiency. In some young patients, CYP7B1 mutations cause severe neonatal liver failure.[28] In this desperate situation, allogenic liver transplantation has been performed to avert the life-threatening condition.[29,30] Unfortunately, no data are available on the long-term outcome of the patients who have undergone liver transplantation. Personal inquiries to the authors of the liver transplant studies have not been successful. The oldest patient would now be well into his teens, and at an age when SPG5 typically becomes symptomatic. It would be of great interest to know his neurological status and his oxysterol levels in CSF. Based on the data from our study, even the highly invasive procedure of liver transplantation is unlikely to prevent the development of an SPG5 phenotype in these patients. The same is true for a potential liver-specific gene therapy for the treatment of severe neonatal liver failure in homozygous CYP7B1 mutation carriers.

The notion that CYP7B1 expression in the CNS is required for a healthy state of the brain and spinal cord is supported by the fact that CYP7B1 physiologically shows substantial expression in oligodendrocytes.[31] This expression pattern may protect the cell type that forms axon sheets and stabilizes long axon tracts from the incorporation of oxysterols and prevent the lipophilic membrane structures from instability and disruption.

Blood levels of the cerebral cholesterol metabolite 24-HC remained unaffected throughout the study, whereas a slight reduction of 24-HC was observed in the liver at the highest doses of AAV8-TTR-hCYP7B1. This may imply that *CYP7B1* can use 24-HC as a substrate, at least to a small extent. The slight elevation of 24-HC in the brain of animals receiving the highest dose of AAV-TTR-hCYP7B1, although significant when compared to the vehicle group, is within the range of untreated KO animals and most likely not correlated to the treatment. This indicates a high specificity of the CYP7B1 treatment with effects limited to the intended metabolites. This is of major importance because 24-HC is the main regulator of cholesterol metabolism in the brain.

In conclusion, this study demonstrated that intravenous treatment with AAV8-TTR-hCYP7B1 leads to a rapid and durable normalization of the metabolic abnormalities in the blood and liver of an SPG5 mouse model. While the expression of *CYP7B1* in the liver by far

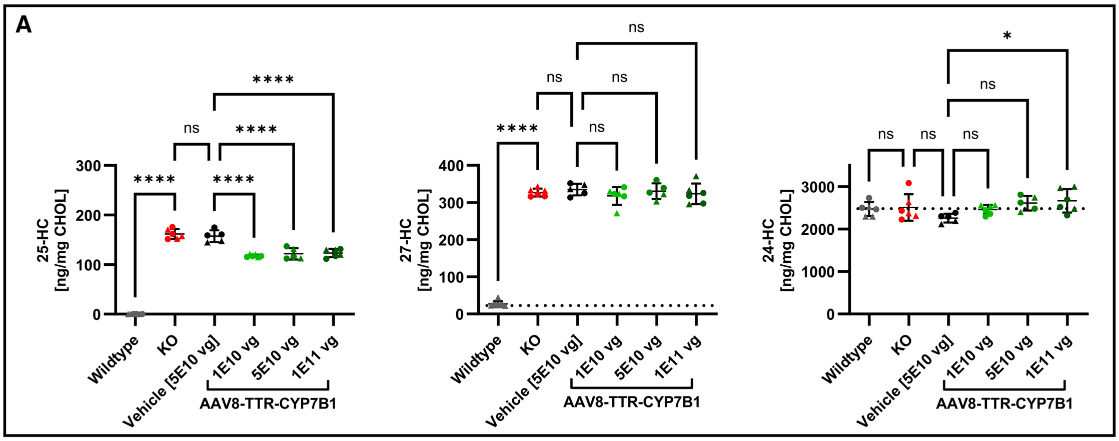

Figure 5. Oxysterol levels in the brain at endpoint

(A) Oxysterol biomarker concentrations in brain homogenate. While a significant reduction of 25-HC was achieved in all treatment dosages, the levels did not come close to WT levels. No effect on brain 27-HC levels could be detected. Levels of the cerebral metabolite 24-HC were not altered except for a slight elevation in the highest dose, within range of the KO group. Triangular points represent data from male, circles from female animals. ns, not significant; *$p < 0.05$; **$p < 0.01$; ***$p < 0.001$; and ****$p < 0.0001$.

exceeded WT levels, the treatment showed a good safety profile with no apparent toxicity. The persistently high levels of key oxysterols in the CNS after liver treatment suggest that a therapeutic approach in SPG5 will most likely need to target the brain as well. Recently published capsid variants derived from AAV9 offer a promising tool to test this hypothesis, as they are reported to transduce not only the liver but also the brain with high efficiency after intravenous application.[32]

MATERIALS AND METHODS

Plasmid cloning

The plasmid pDS-AAV-TTR-Cre (kindly provided by Axel Schambach, Hannover Medical School (MHH)) was used as vector backbone and contained self-complementary inverted terminal repeats of AAV serotype-2 (scITR2) and the Cre-recombinase cDNA under the control of the liver-specific minimal transthyretin promoter (TTR_{min}) and human elongation factor EF-1α intron. The human CYP7B1 cDNA (*hCYP7B1*, GenBank AF127090.1) fused to a Myc-FLAG-tag under the control of a CMV promoter/enhancer was obtained from OriGene. To generate pDS-AAV-TTR-CYP7B1 and pDS-AAV-TTR-CYP7B1-Myc-FLAG-tag, hCYP7B1 and hCYP7B1-Myc-FLAG-tag were PCR amplified and digested with NotI-HF (NEB) and XbaI (NEB). pDS-AAV-TTR-Cre was digested using the same restriction enzymes to yield pDS-AAV-TTR vector backbone, ligated to the digested PCR fragments using T4 Ligase (NEB) and transformed in NEB Stable *E. coli*.

Virus production

Recombinant AAV packaging a self-complementary genome containing GFP or hCYP7B1 cDNA under the control of TTR_{min} were produced and purified as described.[33,34] Briefly, plasmids pDP8.ape (PlasmidFactory GmbH, containing ITR-free *rep2*/*cap8* and adenoviral helper genes) and the plasmid encoding the scITR2-flanked AAV genome were co-transfected in HEK293 using polyethylenimine (PEI Max, Polysciences). Seventy-two hours post-transfection, supernatant and cells were harvested. Cells were lysed by four consecutive freeze-thaw cycles and genomic DNA was degraded by Benzonase digest. rAAV-8 in supernatant and cell lysate were then precipitated as described using 8% (w/v) PEG-8000 and 0.1 M NaCl at 4°C overnight.[35] Resuspended pellet was again treated with Benzonase and then purified by discontinuous iodixanol (Optiprep, Merck) gradient centrifugation.[34] rAAV-8 were concentrated and buffer was exchanged to PBS by centrifugal filtration (100 kDa MWCO, Merck). Final solution was sterile filtrated through a 0.22 μm filter.

To determine viral genomic titer per ml (vg/mL), rAAV8 were lysed in 1 M NaOH at 56°C for 30 min. The pH was subsequently neutralized with 1 M HCl solution and vg/mL was determined by qPCR using TTR_{min} specific primers (FWD: 5′-TCAGCTTGGCAGGGAT CAG-3′ and REV: 5′-GACGGCTTCTCCTGGTGAAG-3′) and SYBR Green Universal Master Mix (Thermo Fisher Scientific).

RT-qPCR

RNA from cultured cells was isolated using the RNeasy mini spin kit (Thermo Fisher Scientific) according to manufacturer's instructions. RNA from liver was isolated using the AllPrep DNA/RNA/Protein Mini kit (QIAGEN) and reverse transcribed using the RevertAid first strand cDNA synthesis kit (Thermo Fisher Scientific) according to manufacturer's instructions. An intron spanning primer pair against human *CYP7B1* (Primer sequences (5′-3′) FWD: CAGTTCTTC TTGGTGGAAAGTA and REV: TGCAACTGACTGATGCTAAA) and mouse *cyp7b1* (FWD: GACGATCCTGAAATAGGAGCACA and REV: AATGGTGTTTGCTAGAGAGGCC) was used to determine the relative expression of the endogenous gene and the transduced construct. All measurements of cultured cells were normalized

to the housekeeping gene GAPDH (FWD: TCACCAGGGCTGC TTTTAAC and REV: GACAAGCTTCCCGTTCTCAG) and mouse tissue samples to Actβ (FWD: CTAAGGCCAACCGTGAAAAG and REV: ACCAGAGGCATACAGGGACA), a well-established housekeeping gene for mouse liver samples.[36]

Immunofluorescence staining

Cultured cells were fixed with 4% paraformaldehyde (PFA, Thermo Fisher Scientific) for 15 min and subsequently washed with PBS. After permeabilization and blocking with 5% BSA (Sigma Aldrich) and 0.1% Triton X-100 (Carl Roth) in PBS, cells were stained with anti-FLAG (rabbit, 1:500, 14793S, Cell Signaling Technology) and anti-CLIMP63 (mouse, 1:500, ALX-804-604-C100, Enzo Life Sciences). After an additional washing step, Alexa-Fluor conjugated secondary antibodies (1:1000, Invitrogen) were applied. Nuclei were counter stained using DAPI (1 μg/mL, 62248, Thermo Fisher Scientific). Coverslips were mounted with mounting solution (Agilent Dako). Imaging was performed using the Nikon Ti2e-CSU-W1-SORA spinning disc confocal system with a 60× objective and for detailed view in combination with the 4× SORA magnifier.

Immunohistochemistry staining

Liver tissue samples were fixed in Roti-Histofix 4% at 4°C overnight and then transferred to PBS. After dehydration, the samples were embedded in paraffin. Immunohistochemical staining for CYP7B1 (Proteintech, 24889-1-AP) was performed on 4 μm deparaffinized sections. Sections were incubated in blocking buffer (Avidin/Biotin blocking kit, Vector Labs, SP-2001) and 20% goat serum in 0.1% BSA, with each step lasting 15 min at room temperature. The primary CYP7B1 antibody (1:200) was applied and incubated overnight at 4°C. The biotinylated secondary antibody, goat anti-rabbit (Vector Labs, BA-1000, 1:700), was incubated for 1 h at room temperature. Enzyme complex formation was conducted using the ABC-HRP kit (Vector Labs, PK-4000) for 1 h at room temperature. Color development was achieved using AEC substrate (Dako, K3464), and sections were mounted with Dako Faramount aqueous mounting medium.

Protein isolation and western blot

Cultured cells were harvested by scraping and subsequent centrifugation. The pellet was re-suspended in RIPA buffer (Sigma-Aldrich) with cOmplete mini protease inhibitor cocktail (PI) (Roche), incubated at 4°C for 45 min and insoluble residues separated by centrifugation (10,000 rcf, 30 min, 4°C). Liver protein samples were isolated by addition of 4 μL RIPA buffer (with PI) per mg of tissue and homogenized using a bead-based tissue homogenizing system (Percellys). After lysis, the homogenate was centrifuged (10,000 rcf, 30 min, 4°C), supernatant was collected and frozen at −80°C in aliquots for western blot. The total protein content was analyzed by using Pierce BCA protein assay kit (Thermo Fisher Scientific) according to manufacturer's instructions. For tissue homogenates, 30 mg of total protein was loaded (10 mg for cultured cells), separated by SDS-PAGE and blotted onto nitrocellulose membranes (Odyssey nitrocellulose membrane 0.22 mm, LI-COR Biosciences), followed by blocking with 5% skimmed milk in Tris-buffered saline (TBS) containing 0.1% Tween 20 buffer (TBST) (Sigma-Aldrich) for 2 h. Subsequently, the membranes were incubated overnight with anti-vinculin antibody (Merck; mouse, 1:100,000, V9131) or anti-*CYP7B1* antibody (Abcam; rabbit, 1:1,000, ab138497) diluted in Roche block solution (Roche, Switzerland). Membranes were washed three times with TBST and incubated for 1 h with an HRP-conjugated secondary antibody against rabbit (AffiniPure goat anti-rabbit, 1:10,000 Jackson ImmunoResearch) or mouse (AffiniPure goat anti-mouse, 1:10,000 Jackson ImmunoResearch) each diluted in 0.5% skimmed milk in TBST. Membranes were washed three times each for 10 min in TBST and stored in TBS until analysis. Protein detection and image processing was carried out with the Gel Doc XR+ system (Bio-Rad).

Animals

All research and animal care procedures were approved by the district government (Regierungspräsidium Tübingen, Germany) and performed according to international guidelines for the use of laboratory animals in the FORS animal facility (Tübingen, Germany). The SPG5 mouse model (*Cyp7b1*$^{-/-}$) used in this study was established by Jan-Åke Gustafsson[19,37] and has been used previously in our laboratory for mRNA-based studies.[20] Animals did not display any obvious motor symptoms, but recapitulate the metabolic phenotype of elevated oxysterols. Mice were housed in standard laboratory cages (type II L) in temperature (22°C ± 2°C) and humidity-controlled (55% ± 10%) rooms. Mice were kept on a 12 h light/dark cycle and had *ad libitum* access to food and water. *Cyp7b1*$^{-/-}$ mice (C57BL/6J background) between 5 and 6 months of age were used in the presented study and male and female animals were evenly distributed in each study group. The AAV preparations were diluted in sterile PBS and injected into the tail vein of the animals at dosages of 1 × 10E10, 5 × 10E10, and 1 × 10E10 vg for AAV8-TTR-hCYP7B1 or 5 × 10E10 vg for AAV8-TTR-eGFP. Control groups received the same volume of sterile PBS, but without AAV. Blood samples were collected by retro bulbar blood draw seven days prior and two days after virus administration for assessment of oxysterol baseline levels and short-term effects on oxysterol concentrations after virus administration. Blood samples from the same experimental group and sex were pooled. Serum was separated by centrifugation (10,000 rcf, 5 min) using serum Gel Z tubes (Sarstedt, Germany), frozen immediately on dry ice and stored at −80°C upon measurement. Animals were sacrificed six weeks after viral vector administration by intraperitoneal injection of Ketamin/Xylazin, heart puncture and subsequent perfusion. Tissues were harvested and frozen or prepared for IHC/immunofluorescence analysis.

Measurement of aminotransferases

Endpoint serum samples were diluted 1:4 in 0.8% (w:v) saline solution and analyzed for AST and ALT levels (IU/L) using an Olympus AU 400 analyzer (Beckman Coulter).

Oxysterol quantification by isotope dilution HPLC-MS

Oxysterol levels were assessed by isotope dilution mass spectrometry using deuterium-labelled internal standards as described

previously.[38,39] Values of oxysterols were normalized to total cholesterol content (ng/mg CHOL).

Statistics

GraphPad Prism 9 software has been used for statistical analysis. Group comparison of oxysterol levels has been performed by ordinary one-way ANOVA analysis. Selected pairwise comparisons are shown in the graphs (*$p < 0.05$, **$p < 0.01$, ***$p < 0.001$, and ****$p < 0.0001$). All error bars depicted in this publication refer to SD.

DATA AVAILABILITY

All raw data presented in this study are available upon reasonable request by the lead contact, Stefan Hauser (stefan.hauser@dzne.de) and Ludger Schöls (ludger.schoels@uni-tuebingen.de).

ACKNOWLEDGMENTS

This study has been supported by the German Research Foundation (DFG, grants SCHO754/8-1, HA9848/2-1, and OT131/11-1). We are grateful to Axel Schambach (MHH, Hannover) for providing the TTR_{min} promotor and the vector backbone. We like to thank Ulrike Naumann (HIH, Tübingen) for assistance and advice in animal experiments. We acknowledge support by Open Access Publishing Fund of University of Tübingen.

AUTHOR CONTRIBUTIONS

The study has been designed by L.W., L.S., S. Hauser, and M.O. AAV cloning and production has been performed by Q.Y. and S. Hook. *In vitro* testing of AAV plasmids has been done by L.W. Animal experiments including blood sampling, vector administration, sacrificing, and organ sampling has been performed by L.W., M.K., and S. Hauser. Gene expression analysis by qPCR and western blot has been performed by L.W. Tissue sectioning and subsequent IHC and immunofluorescence stainings were done by Q.Y. Oxysterol levels have been assessed by I.B. The manuscript has been prepared by L. W., L.S., and S. Hauser. All authors have reviewed and approved the manuscript.

Conceptualization, L.W., M.O., S. Hauser, and L.S.; methodology, L.W., M.O., S. Hauser, and L.S.; formal analysis, L.W., I.B., and S. Hauser; investigation, L.W., Q.Y., S. Hook, M. K., I.B., and S. Hauser; data curation, M.O., S. Hauser, and L.S.; writing – original draft, L.W. and S. Hauser; writing – review & editing, Q.Y., S. Hook, M.O., and L.S.; visualization, L.W.; supervision, M.O., S. Hauser, and L.S.; funding acquisition, M.O., S. Hauser, and L.S.

DECLARATION OF INTERESTS

The authors declare no competing interests.

SUPPLEMENTAL INFORMATION

Supplemental information can be found online at https://doi.org/10.1016/j.omtm.2025.101531.

REFERENCES

1. Schüle, R., and Schöls, L. (2011). Genetics of hereditary spastic paraplegias. Semin. Neurol. *31*, 484–493. https://doi.org/10.1055/s-0031-1299787.
2. Hedera P. Hereditary Spastic Paraplegia Overview. 2019. https://www.ncbi.nlm.nih.gov/books/NBK1509/.
3. Züchner, S., Wang, G., Tran-Viet, K.N., Nance, M.A., Gaskell, P.C., Vance, J.M., Ashley-Koch, A.E., and Pericak-Vance, M.A. (2006). Mutations in the novel mitochondrial protein REEP1 cause hereditary spastic paraplegia type 31. Am. J. Hum. Genet. *79*, 365–369. https://doi.org/10.1086/505361.
4. Solowska, J.M., and Baas, P.W. (2015). Hereditary spastic paraplegia SPG4: What is known and not known about the disease. Brain *138*, 2471–2484. https://doi.org/10.1093/brain/awv178.
5. Gabrych, D.R., Lau, V.Z., Niwa, S., and Silverman, M.A. (2019). Going Too Far Is the Same as Falling Short†: Kinesin-3 Family Members in Hereditary Spastic Paraplegia. Front. Cell. Neurosci. *13*, 419–424. https://doi.org/10.3389/fncel.2019.00419.
6. Schöls, L., Rattay, T.W., Martus, P., Meisner, C., Baets, J., Fischer, I., Jägle, C., Fraidakis, M.J., Martinuzzi, A., Saute, J.A., et al. (2017). Hereditary spastic paraplegia type 5: Natural history, biomarkers and a randomized controlled trial. Brain *140*, 3112–3127. https://doi.org/10.1093/brain/awx273.
7. Goizet, C., Boukhris, A., Durr, A., Beetz, C., Truchetto, J., Tesson, C., Tsaousidou, M., Forlani, S., Guyant-Maréchal, L., Fontaine, B., et al. (2009). CYP7B1 mutations in pure and complex forms of hereditary spastic paraplegia type 5. Brain *132*, 1589–1600. https://doi.org/10.1093/brain/awp073.
8. Tsaousidou, M.K., Ouahchi, K., Warner, T.T., Yang, Y., Simpson, M.A., Laing, N.G., Wilkinson, P.A., Madrid, R.E., Patel, H., Hentati, F., et al. (2008). Sequence Alterations within CYP7B1 Implicate Defective Cholesterol Homeostasis in Motor-Neuron Degeneration. Am. J. Hum. Genet. *82*, 510–515. https://doi.org/10.1016/j.ajhg.2007.10.001.
9. Pikuleva, I.A., Babiker, A., Waterman, M.R., and Björkhem, I. (1998). Activities of recombinant human cytochrome P450c27 (CYP27) which produce intermediates of alternative bile acid biosynthetic pathways. J. Biol. Chem. *273*, 18153–18160. https://doi.org/10.1074/jbc.273.29.18153.
10. Marelli, C., Salsano, E., Politi, L.S., and Labauge, P. (2019). Spinal cord involvement in adult-onset metabolic and genetic diseases. J. Neurol. Neurosurg. Psychiatry *90*, 211–218. https://doi.org/10.1136/jnnp-2018-318666.
11. Schüle, R., Siddique, T., Deng, H.X., Yang, Y., Donkervoort, S., Hansson, M., Madrid, R.E., Siddique, N., Schöls, L., and Björkhem, I. (2010). Marked accumulation of 27-hydroxycholesterol in SPG5 patients with hereditary spastic paresis. J. Lipid Res. *51*, 819–823. https://doi.org/10.1194/jlr.M002543.
12. Björkhem, I., Leoni, V., and Svenningsson, P. (2019). On the fluxes of side-chain oxidized oxysterols across blood-brain and blood-CSF barriers and origin of these steroids in CSF (Review). J. Steroid Biochem. Mol. Biol. *188*, 86–89. https://doi.org/10.1016/j.jsbmb.2018.12.009.
13. Porru, E., Edström, E., Saeed, A.A., Eggertsen, G., Lövgren-Sandblom, A., Roda, A., and Björkhem, I. (2020). Further evidence for a continuous flux of bile acids into the brain: trapping of bile acids in subdural hematomas. Scand. J. Clin. Lab. Invest. *80*, 395–400. https://doi.org/10.1080/00365513.2020.1753108.
14. Heverin, M., Meaney, S., Lütjohann, D., Diczfalusy, U., Wahren, J., and Björkhem, I. (2005). Crossing the barrier: Net flux of 27-hydroxycholesterol into the human brain. J. Lipid Res. *46*, 1047–1052. https://doi.org/10.1194/jlr.M500024-JLR200.
15. Heverin, M., Bogdanovic, N., Lütjohann, D., Bayer, T., Pikuleva, I., Bretillon, L., Diczfalusy, U., Winblad, B., and Björkhem, I. (2004). Changes in the levels of cerebral and extracerebral sterols in the brain of patients with Alzheimer's disease. J. Lipid Res. *45*, 186–193. https://doi.org/10.1194/jlr.M300320-JLR200.
16. Lim, W.L.F., Martins, I.J., and Martins, R.N. (2014). The involvement of lipids in alzheimer's disease. J. Genet. Genomics *41*, 261–274. https://doi.org/10.1016/j.jgg.2014.04.003.
17. Marelli, C., Lamari, F., Rainteau, D., Lafourcade, A., Banneau, G., Humbert, L., Monin, M.L., Petit, E., Debs, R., Castelnovo, G., et al. (2018). Plasma oxysterols: biomarkers for diagnosis and treatment in spastic paraplegia type 5. Brain *141*, 72–84. https://doi.org/10.1093/brain/awx297.
18. Mignarri, A., Malandrini, A., Del Puppo, M., Magni, A., Monti, L., Ginanneschi, F., Tessa, A., Santorelli, F.M., Federico, A., and Dotti, M.T. (2015). Treatment of SPG5 with cholesterol-lowering drugs. J. Neurol. *262*, 2783–2785. https://doi.org/10.1007/s00415-015-7971-5.
19. Li-hawkins, J., Lund, E.G., Turley, S.D., and Russell, D.W. (2000). Disruption of the Oxysterol 7 -Hydroxylase Gene in Mice. J. Biol. Chem. *275*, 16536–16542. https://doi.org/10.1074/jbc.M001811200.
20. Hauser, S., Poenisch, M., Schelling, Y., Höflinger, P., Schuster, S., Teegler, A., Betten, R., Gustafsson, J.Å., Hübener-Schmid, J., Schlake, T., et al. (2019). mRNA as a Novel Treatment Strategy for Hereditary Spastic Paraplegia Type 5. Mol. Ther. Methods Clin. Dev. *15*, 359–370. https://doi.org/10.1016/j.omtm.2019.10.011.
21. Mendell, J.R., Al-Zaidy, S., Shell, R., Arnold, W.D., Rodino-Klapac, L.R., Prior, T.W., Lowes, L., Alfano, L., Berry, K., Church, K., et al. (2017). Single-Dose

Gene-Replacement Therapy for Spinal Muscular Atrophy. N. Engl. J. Med. *377*, 1713–1722. https://doi.org/10.1056/nejmoa1706198.

22. Nathwani, A.C., Reiss, U.M., Tuddenham, E.G.D., Rosales, C., Chowdary, P., McIntosh, J., Della Peruta, M., Lheriteau, E., Patel, N., Raj, D., et al. (2014). Long-Term Safety and Efficacy of Factor IX Gene Therapy in Hemophilia B. N. Engl. J. Med. *371*, 1994–2004. https://doi.org/10.1056/nejmoa1407309.

23. Junge, N., Mingozzi, F., Ott, M., and Baumann, U. (2015). Adeno-Associated Virus Vector – Based Gene Therapy for Monogenetic Metabolic Diseases of the Liver. J. Pediatr. Gastroenterol. Nutr. *60*, 433–440. https://doi.org/10.1097/MPG.0000000000000703.

24. Zabaleta, N., Unzu, C., Weber, N.D., and Gonzalez-Aseguinolaza, G. (2023). Gene therapy for liver diseases — progress and challenges. Nat. Rev. Gastroenterol. Hepatol. *20*, 288–305. https://doi.org/10.1038/s41575-022-00729-0.

25. Yantsevich, A.V., Dichenko, Y.V., Mackenzie, F., Mukha, D.V., Baranovsky, A.V., Gilep, A.A., Usanov, S.A., and Strushkevich, N.V. (2014). Human steroid and oxysterol 7α-hydroxylase CYP7B1: Substrate specificity, azole binding and misfolding of clinically relevant mutants. FEBS J. *281*, 1700–1713. https://doi.org/10.1111/febs.12733.

26. Hilsch, M., Haralampiev, I., Müller, P., Huster, D., and Scheidt, H.A. (2017). Membrane properties of hydroxycholesterols related to the brain cholesterol metabolism. Beilstein J. Org. Chem. *13*, 720–727. https://doi.org/10.3762/bjoc.13.71.

27. Griffiths, W.J., and Wang, Y. (2020). Oxysterols as lipid mediators: Their biosynthetic genes, enzymes and metabolites. Prostaglandins Other Lipid Mediat. *147*, 106381. https://doi.org/10.1016/j.prostaglandins.2019.106381.

28. Setchell, K.D., Schwarz, M., O'Connell, N.C., Lund, E.G., Davis, D.L., Lathe, R., Thompson, H.R., Weslie Tyson, R., Sokol, R.J., and Russell, D.W. (1998). Identification of a new inborn error in bile acid synthesis: Mutation of the oxysterol 7α-hydroxylase gene causes severe neonatal liver disease. J. Clin. Investig. *102*, 1690–1703. https://doi.org/10.1172/JCI2962.

29. Hong, J., Oh, S.H., Yoo, H.W., Nittono, H., Kimura, A., and Kim, K.M. (2018). Complete recovery of oxysterol 7α-hydroxylase deficiency by living donor transplantation in a 4-month-old infant: The first Korean case report and literature review. J. Korean Med. Sci. *33*, e324. https://doi.org/10.3346/jkms.2018.33.e324.

30. Mizuochi, T., Kimura, A., Suzuki, M., Ueki, I., Takei, H., Nittono, H., Kakiuchi, T., Shigeta, T., Sakamoto, S., Fukuda, A., et al. (2011). Successful heterozygous living donor liver transplantation for an oxysterol 7α-hydroxylase deficiency in a Japanese patient: LT for Mutations of the *CYP7B1* Gene. Liver Transpl. *17*, 1059–1065. https://doi.org/10.1002/lt.22331.

31. Karlsson, M., Zhang, C., Méar, L., Zhong, W., Uhlén, M., and Lindskog, C. (2021). A single–cell type transcriptomics map of human tissues. Sci. Adv. *7*, eabh2169. https://doi.org/10.1126/sciadv.abh2169.

32. Goertsen, D., Flytzanis, N.C., Goeden, N., Chuapoco, M.R., Cummins, A., Chen, Y., Fan, Y., Zhang, Q., Sharma, J., Duan, Y., et al. (2022). AAV capsid variants with brain-wide transgene expression and decreased liver targeting after intravenous delivery in mouse and marmoset. Nat. Neurosci. *25*, 106–115. https://doi.org/10.1038/s41593-021-00969-4.

33. Grimm, D., Kay, M.A., and Kleinschmidt, J.A. (2003). Helper virus-free, optically controllable, and two-plasmid-based production of adeno-associated virus vectors of serotypes 1 to 6. Mol. Ther. *7*, 839–850. https://doi.org/10.1016/S1525-0016(03)00095-9.

34. Zolotukhin, S., Byrne, B.J., Mason, E., Zolotukhin, I., Potter, M., Chesnut, K., Summerford, C., Samulski, R.J., and Muzyczka, N. (1999). Recombinant adeno-associated virus purification using novel methods improves infectious titer and yield. Gene Ther. *6*, 973–985. https://doi.org/10.1038/sj.gt.3300938.

35. Guo, P., El-Gohary, Y., Prasadan, K., Shiota, C., Xiao, X., Wiersch, J., Paredes, J., Tulachan, S., and Gittes, G.K. (2012). Rapid and simplified purification of recombinant adeno-associated virus. J. Virol. Methods *183*, 139–146. https://doi.org/10.1016/j.jviromet.2012.04.004.

36. Day, P.E.L., Chambers, K.F., Winterbone, M.S., García-Blanco, T., Vauzour, D., and Kroon, P.A. (2018). Validation of control genes and a standardised protocol for quantifying gene expression in the livers of C57BL/6 and ApoE-/-mice. Sci. Rep. *8*, 8081. https://doi.org/10.1038/s41598-018-26431-3.

37. Rose, K., Allan, A., Gauldie, S., Stapleton, G., Dobbie, L., Dott, K., Martin, C., Wang, L., Hedlund, E., Seckl, J.R., et al. (2001). Neurosteroid hydroxylase CYP7B: Vivid reporter activity in dentate gyrus of gene-targeted mice and abolition of a widespread pathway of steroid and oxysterol hydroxylation. J. Biol. Chem. *276*, 23937–23944. https://doi.org/10.1074/jbc.M011564200.

38. Dzeletovic, S., Breuer, O., Lind, E., and Diczfalusy, U. (1994). Determination of Cholesterol Oxidation Products in Human Plasma by Isotope Dilution-Mass Spectrometry. Anal. Biochem. *225*, 73–80. https://doi.org/10.1006/abio.1995.1110.

39. Porru, E., Edström, E., Arvidsson, L., Elmi-Terander, A., Fletcher-Sandersjöö, A., Sandblom, A.L., Hansson, M., Duell, F., and Björkhem, I. (2022). Ventriculoperitoneal Shunt Treatment Increases 7 Alpha Hy-Droxy-3-Oxo-4-Cholestenoic Acid and 24-Hydroxycholesterol Concentrations in Idiopathic Normal Pressure Hydrocephalus. Brain Sci. *12*, 1450. https://doi.org/10.3390/brainsci12111450.

OMTM, Volume *33*

Supplemental information

AAV8-based gene replacement therapy for hereditary spastic paraplegia type 5

Linus Wiora, Qinggong Yuan, Sebastian Hook, Melanie Kraft, Ingemar Björkhem, Michael Ott, Stefan Hauser, and Ludger Schöls

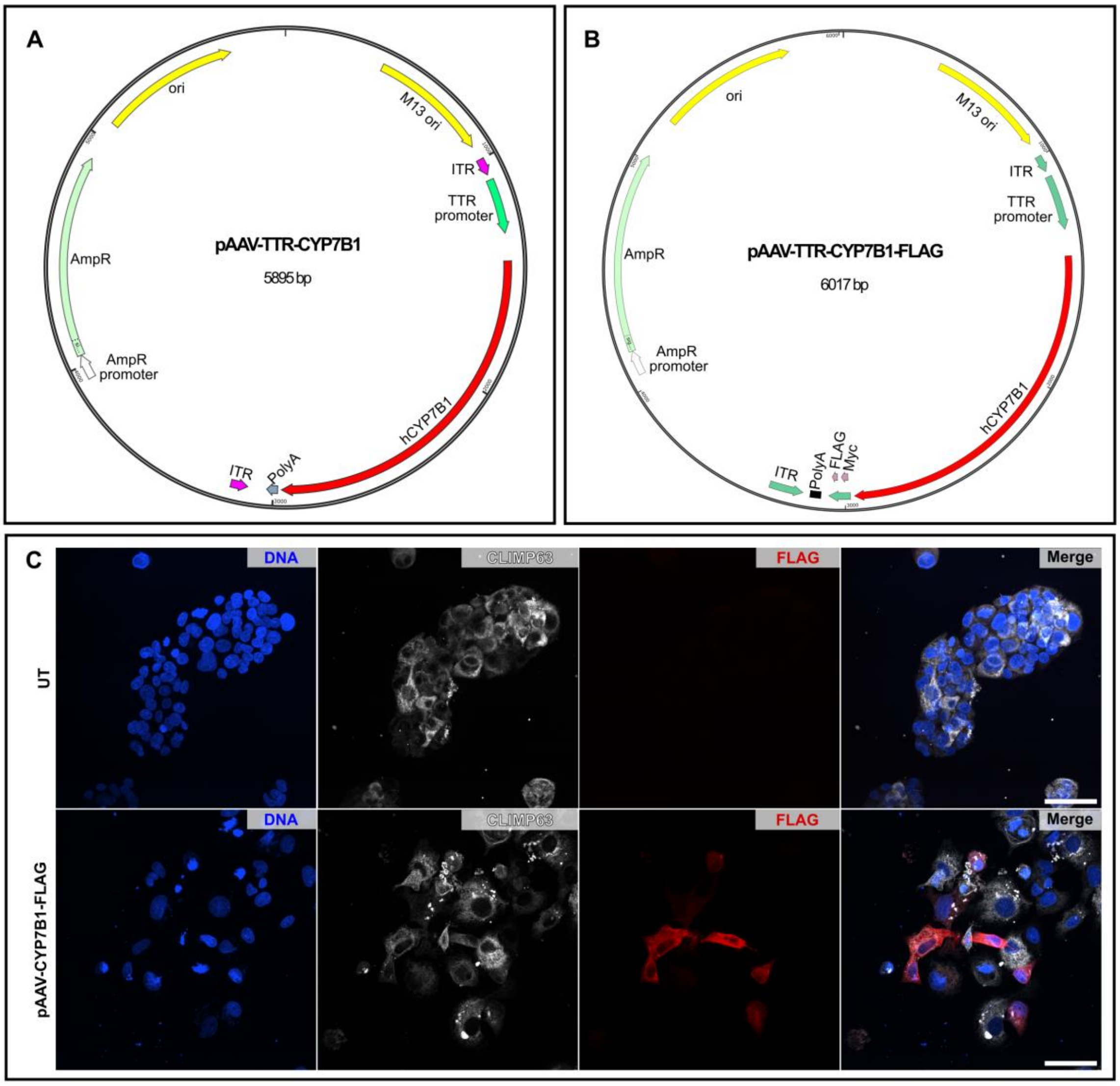

Figure S1: Plasmids and specificity test for FLAG antibody. Plasmid maps of (A) pAAV-TTR-hCYP7B1 and (B) pAAV-TTR-CYP7B1-FLAG construct used for expression test and virus production. (C) HepG2 cells immunostained for the FLAG-tag. Untreated control (UT) shows only signal for the endogenous ER marker CLIMP63, while cells transduced with pAAV-TTR-CYP7B1-FLAG show clear signal localizing in the ER with CLIMP63, demonstrating specificity of the antibody. Scale bar= 50 µm.
